# Intermittent explosive disorder subtypes in the general population: association with comorbidity, impairment and suicidality

**DOI:** 10.1017/S2045796020000517

**Published:** 2020-06-23

**Authors:** K. M. Scott, Y. A. de Vries, S. Aguilar-Gaxiola, A. Al-Hamzawi, J. Alonso, E. J. Bromet, B. Bunting, J. M. Caldas-de-Almeida, A. Cía, S. Florescu, O. Gureje, C-Y. Hu, E. G. Karam, A. Karam, N. Kawakami, R. C. Kessler, S. Lee, J. McGrath, B. Oladeji, J. Posada-Villa, D. J. Stein, Z. Zarkov, P. de Jonge

**Affiliations:** 1 Department of Psychological Medicine, Dunedin School of Medicine, University of Otago, PO Box 56, Dunedin 9054, New Zealand; 2 Department of Developmental Psychology, Rijksuniversiteit Groningen, Groningen, Netherlands; 3 Department of Psychiatry, Interdisciplinary Center Psychopathology and Emotion Regulation, University Medical Center Groningen, Groningen, Netherlands; 4 Center for Reducing Health Disparities, UC Davis Health System, Sacramento, California, USA; 5 College of Medicine, Al-Qadisiya University, Diwaniya governorate, Iraq; 6 Health Services Research Unit, IMIM-Hospital del Mar Medical Research Institute, Barcelona, Spain; 7 CIBER en Epidemiología y Salud Pública (CIBERESP), Barcelona, Spain; 8 Pompeu Fabra University (UPF), Barcelona, Spain; 9 Department of Psychiatry, Stony Brook University School of Medicine, Stony Brook, New York, USA; 10 School of Psychology, Ulster University, Londonderry, UK; 11 Lisbon Institute of Global Mental Health and Chronic Diseases Research Center (CEDOC), NOVA Medical School | Faculdade de Ciências Médicas, Universidade Nova de Lisboa, Lisbon, Portugal; 12 Anxiety Disorders Center, Buenos Aires, Argentina; 13 National School of Public Health, Management and Development, Bucharest, Romania; 14 Department of Psychiatry, University College Hospital, Ibadan, Nigeria; 15 Shenzhen Institute of Mental Health & Shenzhen Kangning Hospital, Shenzhen, China; 16 Department of Psychiatry and Clinical Psychology, Faculty of Medicine, Balamand University, Beirut, Lebanon; 17 Department of Psychiatry and Clinical Psychology, St George Hospital University Medical Center, Beirut, Lebanon; 18 Institute for Development Research Advocacy and Applied Care (IDRAAC), Beirut, Lebanon; 19 Department of Mental Health, School of Public Health, The University of Tokyo, Tokyo, Japan; 20 Department of Health Care Policy, Harvard Medical School, Boston, Massachusetts, USA; 21 Department of Psychiatry, Chinese University of Hong Kong, Tai Po, Hong Kong; 22 Queensland Centre for Mental Health Research, The Park Centre for Mental Health, Wacol QLD 4072, Australia; 23 Queensland Brain Institute, The University of Queensland, St Lucia QLD 4065, Australia; 24 National Centre for Register-based Research, Aarhus University, Aarhus V 8000, Denmark; 25 Department of Psychiatry, College of Medicine, University of Ibadan; University College Hospital, Ibadan, Nigeria (Bibiloba); 26 Faculty of Social Sciences, Colegio Mayor de Cundinamarca University, Bogota, Colombia; 27 Department of Psychiatry & Mental Health and South African Medical Council Research Unit on Risk and Resilience in Mental Disorders, University of Cape Town and Groote Schuur Hospital, Cape Town, South Africa; 28 Department of Mental Health, National Center of Public Health and Analyses, Sofia, Bulgaria

**Keywords:** Comorbidity, impairment, Intermittent Explosive Disorder, suicidality, World Mental Health Surveys

## Abstract

**Aims:**

Intermittent explosive disorder (IED) is characterised by impulsive anger attacks that vary greatly across individuals in severity and consequence. Understanding IED subtypes has been limited by lack of large, general population datasets including assessment of IED. Using the 17-country World Mental Health surveys dataset, this study examined whether behavioural subtypes of IED are associated with differing patterns of comorbidity, suicidality and functional impairment.

**Methods:**

IED was assessed using the Composite International Diagnostic Interview in the World Mental Health surveys (*n* = 45 266). Five behavioural subtypes were created based on type of anger attack. Logistic regression assessed association of these subtypes with lifetime comorbidity, lifetime suicidality and 12-month functional impairment.

**Results:**

The lifetime prevalence of IED in all countries was 0.8% (s.e.: 0.0). The two subtypes involving anger attacks that harmed people (‘hurt people only’ and ‘destroy property and hurt people’), collectively comprising 73% of those with IED, were characterised by high rates of externalising comorbid disorders. The remaining three subtypes involving anger attacks that destroyed property only, destroyed property and threatened people, and threatened people only, were characterised by higher rates of internalising than externalising comorbid disorders. Suicidal behaviour did not vary across the five behavioural subtypes but was higher among those with (*v*. those without) comorbid disorders, and among those who perpetrated more violent assaults.

**Conclusions:**

The most common IED behavioural subtypes in these general population samples are associated with high rates of externalising disorders. This contrasts with the findings from clinical studies of IED, which observe a preponderance of internalising disorder comorbidity. This disparity in findings across population and clinical studies, together with the marked heterogeneity that characterises the diagnostic entity of IED, suggests that it is a disorder that requires much greater research.

## Introduction

A prominent bimodal conceptualisation of aggression classifies it as either: (i) spontaneous (referred to as reactive or impulsive aggression), or (ii) planned (referred to as proactive, premediated or instrumental aggression) (Babcock *et al*., 2014; Wrangham, 2018). Impulsive aggression has generally been found to be more characteristic of clinical samples and premeditated aggression more characteristic of delinquent or criminal populations (Jensen *et al*., 2007). The essential feature of intermittent explosive disorder (IED) as defined in both DSM-IV and DSM-5 is the occurrence of repeated episodes of impulsive aggression resulting in verbal or physical assaults or property destruction.

The first population studies on the epidemiology of DSM-IV IED in the USA were undertaken using the World Mental Health (WMH) surveys version of the Composite International Diagnostic Interview (WMH-CIDI) (Kessler *et al*., 2006; McLaughlin *et al*., 2012). In the National Comorbidity Survey Replication (NCS-R), IED prevalence among adults (18 years or older) based on a ‘broad’ definition of IED requiring three or more impulsive anger attacks in the lifetime was estimated to be 7.3%, decreasing to 5.4% based on a ‘narrow’ definition requiring three or more anger attacks in the same year (Kessler *et al*., 2006). In response to reviewer feedback, the WMH-CIDI diagnostic algorithm was subsequently modified to further require that anger attacks should cause at least some degree of interference with respondents' work, social life or relationships, thus bringing the diagnosis of IED into line with the other WMH-CIDI DSM-IV diagnoses. We refer to this revised algorithm as the ‘conservative’ definition of IED, and we applied it in our first cross-national report on IED which found lifetime prevalence ranging across countries from 0.1% to 2.7% with a weighted average of 0.8% (Scott *et al*., 2016, 2018). The sociodemographic correlates of lifetime risk of IED were being male, young, unemployed, divorced or separated and having less education. The median age of onset of IED was 17 and prior traumatic experiences involving physical (non-combat) or sexual violence were associated with increased risk of IED onset (Scott *et al*., 2016, 2018).

That earlier cross-national study focused on IED as a single diagnostic entity; the present study focuses on IED subtypes. It is a notable feature of IED as defined in DSM-IV and DSM-5 that the aggressive outbursts potentially classifiable as IED span a wide spectrum from non-destructive (verbal only) through destruction of property to hurting people. This gives rise to the possibility that IED could be characterised by distinct behavioural subtypes, and although few studies have investigated this, one study did find that comorbidity patterning varied by DSM-5 IED subtypes (verbal aggression only, physical aggression only, or both) (Look *et al*., 2015). In the WMH surveys, we have a sufficiently large number of respondents diagnosed with IED to be able to classify IED subtypes according to the type of aggressive behaviour. In this study, we use the same conservative definition of IED applied in our earlier cross-national report, and we have created five mutually exclusive behavioural subtypes. Our research questions were: (i) whether these behavioural subtypes would be predominantly associated with different types of comorbid mental disorder; and (ii) whether they would vary in associated suicidal behaviour and functional impairment.

## Methods

### Samples and procedures

This study uses data from all WMH surveys that measured IED (online Supplementary Table S1). A stratified multi-stage clustered area probability sampling strategy was used to select adult respondents (18 years+) in most WMH countries. In most countries, internal subsampling was used to reduce respondent burden and average interview time by dividing the interview into two parts. All respondents completed Part 1, which included the core diagnostic assessment of mood disorders, most anxiety disorders, substance use disorders and IED, and also assessed suicidality and sociodemographics. All Part 1 respondents who met lifetime criteria for any mental disorder and a probability sample of respondents without mental disorders were administered Part 2, which assessed post-traumatic stress disorder, eating disorders, childhood impulse-control disorders, psychotic symptoms, physical health, functional impairment, psychological distress, childhood adversities and service use. Part 2 respondents were weighted by the inverse of their probability of selection for Part 2 of the interview to adjust for differential sampling. Additional weights were used to adjust for differential probabilities of selection within households, to adjust for non-response and to match the samples to population sociodemographic distributions. All respondents provided written informed consent and measures taken to ensure data accuracy, cross-national consistency and protection of respondents are described in detail elsewhere (Kessler and Ustun, 2004, 2008).

### Measures

#### Intermittent explosive disorder

All surveys used the WMH survey version of the WHO Composite International Diagnostic Interview (CIDI 3.0) (Kessler and Ustun, 2004), a fully structured, lay-administered, face-to-face interview, to assess lifetime history of DSM-IV mental disorders. DSM-IV Criterion A for IED requires ‘several discrete episodes of failure to resist aggressive impulses that result in serious assaultive acts or destruction of property’. This was operationalised in the CIDI by requiring the respondent to report at least three attacks in the same year of at least one of three types of anger attacks: (i) ‘when all of a sudden you lost control and broke or smashed something worth more than a few dollars’; (ii) ‘when all of a sudden you lost control and hit or tried to hurt someone’; and (iii) ‘when all of a sudden you lost control and threatened to hit or hurt someone’. A 12-month diagnosis was assigned if those meeting lifetime criteria reported at least three attacks in the past 12 months.

DSM-IV criterion B for IED requires that the aggressiveness is ‘grossly out of proportion to any precipitating psychosocial stressor’. This criterion was operationalised in the CIDI by requiring the respondent to report either that they ‘got a lot more angry than most people would have been in the same situation’ or that the attacks occurred ‘without good reason’ or ‘in situations where most people would not have had an anger attack’.

DSM-IV criterion C for IED requires that the ‘aggressive episodes are not better accounted for by another mental disorder and are not due to the direct physiological effects of a substance or a general medical condition’. This was assessed through a series of questions (see (Kessler *et al*., 2006) for details) that ruled out IED diagnosis if anger attacks occurred exclusively when respondents had been drinking or using drugs, when they were in a depressive or manic episode, or as a consequence of an organic cause such as epilepsy, head injury or use of medications.

In this paper, we have applied the conservative definition of IED, requiring that respondents reported that their anger attacks caused at least some degree of interference with their work, social life or relationships. This is the same diagnostic algorithm used in our recent cross-national reports (Scott *et al*., 2016, 2018).

#### DSM-IV *v*. DSM-5 criteria

DSM-5 criteria recognise two different patterns of the aggressive outburst: high frequency/low intensity (criterion A1: non-destructive verbal or physical aggression occurring at least twice weekly for at least three months) or low frequency/high intensity (criterion A2: at least three destructive outbursts within a year-long period) (Coccaro *et al*., 2014). The diagnostic algorithm used in the present study requires three aggressive outbursts within 1 year, but as noted in our earlier paper (Scott *et al*., 2016) there was insufficient information on the lifetime frequency of specific types of aggressive outburst to confirm whether those meeting the DSM-IV criteria operationalised in this study would also meet DSM-5 criteria.

### IED subtypes

IED subtypes were mutually exclusive categories based on the type of behaviour during anger attacks. The CIDI screening questions for IED are based on the assumption that hurting other people is inherently threatening. Therefore, all respondents are asked whether they have ever in their life ‘had attacks of anger when all of a sudden [they] lost control and broke or smashed something worth more than a few dollars’ and whether they have ever ‘had attacks of anger when all of a sudden [they] lost control and hit or tried to hurt someone’. However, only respondents who answer ‘no’ to this second question are asked whether they have ever ‘had attacks of anger when all of a sudden [they] lost control and threatened to hit or hurt someone’. Given this skip logic in the CIDI, the number of possible subtypes is 5. Subtype 1 consisted of people whose anger attacks destroyed property only; subtype 2 consisted of people whose anger attacks threatened people only; subtype 3 consisted of people whose anger attacks hurt people but not property (with or without threatening); subtype 4 destroyed property and threatened people; subtype 5 destroyed property and hurt people.

#### Impairment and suicidality

The assessment of impairment included questions about lifetime impairment as well as impairment in the past 12 months. The lifetime questions were asked about the financial value of all the things the respondent ever broke or damaged during an anger attack and the number of times either the respondent or someone else had to seek medical attention because of an injury caused by one of the respondent's anger attacks. The 12-month questions asked respondents to rate the extent to which their symptoms interfered with their lives and activities in the worst month of the past year using the Sheehan Disability Scales (Leon *et al*., 1997). These are 0–10 visual analogue scales that ask how much a focal disorder interfered with home management, work, social life and personal relationships using the response options none (0), mild (1–3), moderate (4–6), severe (7–10). All respondents were asked whether in their lifetime they had ever seriously thought about committing suicide, and, if so, whether they had ever made a plan or attempted suicide.

### Statistical analysis

Cross-tabulation was used to determine the prevalence of IED and its subtypes. We used logistic regression to examine the association between IED (subtypes) and lifetime comorbidity, lifetime suicidality and 12-month impairment due to IED. Logistic regression was also used to examine the association between the severity of IED-related violence and these outcomes. All analyses controlled for the country of origin of the participant, as well as participant's sex, age and educational attainment. Because the data were clustered and weighted, standard errors were estimated using the Taylor series linearisation method (SUDAAN 11.0.1).

## Results

### Prevalence of IED and its subtypes

Table 1 shows that the overall lifetime prevalence of IED in all countries was 0.8%. The table also shows the prevalence of the five behavioural subtypes. The most common subtype, with a prevalence of 0.4%, was the subtype with the most severe consequences, involving anger attacks that both destroy property and hurt people. The next most common subtype (‘hurt people only’: 0.2%) involves acts of aggression that result in people (but not property) being hurt. The other three subtypes are characterised by acts of aggression that do not result in harm to persons and these were the lowest prevalence, at around 0.1% each.

**Table 1.** Lifetime prevalence of narrow IED (with impairment) and its subtypes

Disorder (subtype)	*n*_1_^a^	*n*_2_^b^	%^c^	s.e.
Intermittent explosive disorder (all subtypes)	651	86 789	0.8	0.04
IED – destroy property only	72	86 789	0.1	0.01
IED – threaten people only	54	86 789	0.1	0.01
IED – hurt people only (with/without threatening)	118	86 789	0.2	0.02
IED – destroy property and threaten people	52	86 789	0.1	0.01
IED – destroy property and hurt people	355	86 789	0.4	0.03

Overall prevalence does not equal the sum of the prevalence of subtypes due to rounding.

Narrow IED is defined as hierarchical IED, with the added criteria that the respondent must have had three or more attacks in a single year at least once and that the respondent reports that the anger attacks interfere with work, social life, or personal relationships to at least some degree.

IED subtypes are based on behaviour during anger attacks. Respondents were asked whether they ever destroyed property or hurt people during an anger attack. If they reported never having hurt someone, they were asked whether they had ever threatened to hurt someone.

a Nominator *N* (number of participants reporting the outcome).

b Denominator *N* (number of participants asked the question).

c Percentages are based on weighted data.

### Lifetime comorbidity

The pattern of lifetime comorbidity among those with IED is shown in Table 2, with people without IED (including those without any disorder) as the reference group. Comorbidity rates were high, with 80.5% of those with IED having at least one comorbid disorder, with anxiety disorders being the largest disorder class (55.1%). The percentages reflect, in part, the base rate of the disorders; the odds ratios provide information about the relative likelihood of specific types of comorbidity disorders after taking the base rate into account. From the column of odds ratios we see that internalising disorders were highly comorbid with IED (OR: 7.4; 95% CI: 5.8–9.5); this reflects the high comorbidity with anxiety disorders as a class (OR: 7.2; 95% CI: 5.8–8.8) and with bipolar disorder (OR: 6.8; 95% CI: 5.1–8.9). But there was not high comorbidity with mood disorders as a class, and indeed depression was the specific disorder with the lowest odds of comorbidity with IED (OR: 2.7; 95% CI: 2.1–3.5).

**Table 2.** Lifetime prevalence of mental disorders in respondents with or without IED

	Respondents without IED				Respondents with IED				Statistical test				
Comorbid disorder	*n*_1_^a^	*n*_2_^b^	%^c^	s.e.	*n*_1_^a^	*n*_2_^b^	%^c^	s.e.	OR	(95% CI)	Wald X2	*p*-value	df
Agoraphobia (with or without panic)	1203	44 651	2.0	0.1	60	615	8.9	1.2	3.7*	(2.7–5.1)	71.2*	<0.001	1
Generalised anxiety disorder	2672	44 651	3.5	0.1	128	615	20.9	2.1	6.6*	(5.0–8.7)	179.4*	<0.001	1
Panic disorder (with or without agoraphobia)	1200	44 651	1.6	0.1	77	615	9.6	1.2	4.6*	(3.4–6.3)	95.9*	<0.001	1
Post-traumatic stress disorder	1889	38 244	3.2	0.1	100	547	16.4	1.7	4.9*	(3.8–6.3)	144.0*	<0.001	1
Separation anxiety disorder	2057	26 309	5.4	0.2	123	433	28.3	2.3	4.5*	(3.5–5.6)	153.8*	<0.001	1
Social phobia	2533	44 651	3.6	0.1	145	615	23.4	2.0	4.9*	(3.9–6.3)	169.9*	<0.001	1
Specific phobia	4560	38 698	7.7	0.2	153	533	26.9	2.1	3.5*	(2.8–4.4)	117.0*	<0.001	1
**Any anxiety disorder**	**8128**	**44 651**	**12.1**	**0.2**	**343**	**615**	**55.1**	**2.3**	7.2*	**(5.8–8.8)**	354.9*	**<0.001**	**1**
Major depression or dysthymia (hierarchical)	7936	44 651	10.2	0.2	190	615	26.4	2.4	2.7*	(2.1–3.5)	59.2*	<0.001	1
Bipolar disorder (broad)	1188	38 698	1.8	0.1	98	533	17.6	1.9	6.8*	(5.1–8.9)	181.4*	<0.001	1
**Any mood disorder**	**9124**	**44 651**	**11.8**	**0.2**	**288**	**615**	**40.8**	**2.7**	4.3*	**(3.4–5.5)**	155.5*	**<0.001**	**1**
Bulimia nervosa	203	17 105	0.7	0.1	12	254	4.4	1.5	5.5*	(2.5–12.1)	18.6*	<0.001	1
Binge eating disorder (hierarchical)	436	17 105	1.8	0.1	17	254	6.5	1.7	3.0*	(1.7–5.2)	15.7*	<0.001	1
**Any eating disorder**	**598**	**17 105**	**2.3**	**0.1**	**29**	**254**	**10.9**	**2.3**	4.2*	**(2.6–6.8)**	35.0*	**<0.001**	**1**
**Any internalising disorder**	**13 487**	**44 651**	**19.0**	**0.3**	**435**	**615**	**67.1**	**2.6**	7.4*	**(5.8–9.5)**	251.7*	**<0.001**	**1**
Attention deficit disorder	589	25 952	1.6	0.1	61	412	16.0	2.5	4.9*	(3.2–7.6)	50.8*	<0.001	1
Conduct disorder	652	21 666	2.2	0.1	84	376	23.8	2.8	6.3*	(4.4–8.8)	111.2*	<0.001	1
Oppositional defiant disorder	775	18 634	2.9	0.2	88	328	26.6	2.9	6.0*	(4.2–8.6)	98.9*	<0.001	1
**Any disruptive behaviour disorder**	**1540**	**28 406**	**3.8**	**0.2**	**150**	**443**	**35.5**	**2.7**	6.9*	**(5.2–9.3)**	173.1*	**<0.001**	**1**
Alcohol abuse	4136	42 226	8.1	0.2	208	565	38.4	2.4	5.6*	(4.5–7.1)	222.4*	<0.001	1
Alcohol dependence	1345	42 226	2.2	0.1	114	565	20.0	2.2	7.0*	(5.0–9.8)	126.5*	<0.001	1
Drug abuse	1236	40 187	2.4	0.1	101	544	17.2	2.0	4.3*	(3.1–6.0)	74.4*	<0.001	1
Drug dependence	447	40 187	0.8	0.1	56	544	9.7	1.4	5.7*	(3.9–8.3)	80.4*	<0.001	1
**Any substance use disorder**	**4703**	**42 226**	**9.2**	**0.2**	**235**	**565**	**43.2**	**2.6**	6.0*	**(4.8–7.6)**	221.4*	**<0.001**	**1**
**Any externalising disorder**	**5629**	**44 651**	**10.2**	**0.2**	**301**	**615**	**50.5**	**2.4**	7.0*	**(5.6–8.8)**	304.7*	**<0.001**	**1**
**Any disorder**	**16 324**	**44 651**	**25.0**	**0.3**	**504**	**615**	**80.5**	**2.2**	10.0*	**(7.5–13.4)**	241.1*	**<0.001**	**1**

Logistic regression was used to compare the prevalence of the comorbid disorder in respondents with IED to that in respondents without IED. All analyses control for participants' age, sex, education (in country-specific quartiles) and country of origin. Bold values highlight the results for disorder classes (i,e. groups of disorders).

a Nominator *N* (number of participants reporting the outcome).

b Denominator *N* (number of participants asked the question).

c Percentages are based on weighted data.

There was also high comorbidity with externalising disorders as a class (OR: 7.0; 95% CI: 5.6–8.8), due in particular to high comorbidity with the class of disruptive behaviour disorders (OR: 6.9; 95% CI: 5.2–9.3) and with alcohol dependence (OR: 7.0; 95% CI: 5.0–9.8).

In analyses that investigated the temporal ordering of IED and comorbid disorders we found that IED first developed following the onset of the comorbid disorder/s in 61% of cases; conversely, IED was the first disorder to occur in only 29% of cases and first developed concurrently with another disorder 10% of the time. (online Supplementary Table S2).

### Lifetime comorbidity by IED subtype

Next, we analysed how comorbidity varied by the five IED subtypes, with ‘No IED’ as the reference group (Table 3). The likelihood of having any comorbid disorder (final row of the table) was highest amongst the subtype whose behaviour resulted in the least destruction ('threaten people only’: OR: 14.6; 95% CI: 5.2–41.3), followed by the subtype whose behaviour resulted in the most destruction (‘destroy property and hurt people’: OR: 11.7; 95% CI: 7.5–18.2). The two subtypes characterised by destruction of property but not harm to people had the least likelihood of a comorbid disorder (‘destroy property and threaten people’: OR: 6.6; 95% CI: 2.0–21.2; and ‘destroy property only’: OR: 6.0; 95% CI: 3.3–11.0).

**Table 3.** Lifetime prevalence of comorbid disorders in respondents with various IED subtypes, compared to those without IED

			Type of IED																						
	No IED		Destroy and hurt				Destroy and threaten				Destroy property				Hurt people				Threaten people				Overall test		
Comorbid disorder	*n*_1_^a^	*n*_2_^b^	*n*_1_^a^	*n*_2_^b^	OR	(95% CI)	*n*_1_^a^	*n*_2_^b^	OR	(95% CI)	*n*_1_^a^	*n*_2_^b^	OR	(95% CI)	*n*_1_^a^	*n*_2_^b^	OR	(95% CI)	*n*_1_^a^	*n*_2_^b^	OR	(95% CI)	Wald X2	*p*-value	df
Agoraphobia (with or without panic)	1203	44 651	41	332	5.0*	(3.3–7.7)	5	51	4.6*	(1.4–14.6)	4	70	3.6*	(1.3–10.3)	3	110	0.8	(0.2–2.8)	7	52	4.9*	(2.0–12.0)	90.6*	<0.001	5
Generalised anxiety disorder	2672	44 651	66	332	6.3*	(4.4–8.8)	15	51	9.2*	(4.9–17.4)	13	70	4.6*	(2.1–10.3)	20	110	7.4*	(3.6–14.8)	14	52	7.1*	(3.2–16.0)	189.2*	<0.001	5
Panic disorder (with or without agoraphobia)	1200	44 651	48	332	5.3*	(3.5–8.1)	9	51	7.7*	(3.1–19.5)	4	70	1.6	(0.6–4.5)	9	110	2.9*	(1.3–6.4)	7	52	4.5*	(1.7–11.6)	101.5*	<0.001	5
Post-traumatic stress disorder	1889	38 244	60	296	5.5*	(4.0–7.8)	11	45	5.4*	(2.5–11.5)	9	60	3.7*	(1.8–7.5)	12	99	3.5*	(1.6–7.4)	8	47	4.8*	(1.7–13.5)	149.4*	<0.001	5
Separation anxiety disorder	2057	26 309	76	248	4.6*	(3.3–6.4)	11	41	4.5*	(1.8–11.1)	11	44	5.7*	(2.7–11.9)	17	65	3.7*	(2.0–7.0)	8	35	3.0*	(1.3–6.9)	162.0*	<0.001	5
Social phobia	2533	44 651	84	332	5.1*	(3.7–7.1)	15	51	4.7*	(2.1–10.3)	13	70	4.2*	(1.9–9.4)	14	110	2.7*	(1.2–6.2)	19	52	13.3*	(7.0–25.1)	197.3*	<0.001	5
Specific phobia	4560	38 698	92	299	4.1*	(3.0–5.7)	20	50	5.8*	(2.9–11.4)	13	60	1.9	(0.9–4.2)	17	83	1.7	(0.9–3.2)	11	41	3.4*	(1.4–8.3)	129.0*	<0.001	5
**Any anxiety disorder**	**8128**	**44 651**	**199**	**332**	8.7*	**(6.4–11.9)**	**30**	**51**	6.1*	**(2.7–14.0)**	**35**	**70**	6.5*	**(3.6–11.9)**	**48**	**110**	4.6*	**(2.6–8.4)**	**31**	**52**	9.4*	**(4.9–18.0)**	365.2*	**<0.001**	**5**
Major depression or dysthymia (hierarchical)	7936	44 651	109	332	2.9*	(2.0–4.1)	15	51	2.6*	(1.3–5.3)	21	70	2.8*	(1.3–6.4)	26	110	1.8*	(1.1–3.1)	19	52	4.1*	(2.0–8.4)	65.1*	<0.001	5
Bipolar disorder (broad)	1188	38 698	62	299	6.9*	(5.0–9.6)	11	50	10.0*	(4.5–22.5)	7	60	3.7*	(1.5–8.9)	11	83	3.9*	(1.8–8.5)	7	41	11.3*	(3.2–39.9)	210.7*	<0.001	5
**Any mood disorder**	**9124**	**44 651**	**171**	**332**	4.9*	**(3.7–6.6)**	**26**	**51**	6.0*	**(3.0–11.8)**	**28**	**70**	3.4*	**(1.6–7.0)**	**37**	**110**	2.2*	**(1.4–3.5)**	**26**	**52**	7.9*	**(2.9–21.4)**	158.1*	**<0.001**	**5**
Bulimia nervosa	203	17 105	6	141	4.1*	(1.4–11.7)	3	23	23.8*	(6.1–93.2)	1	29	5.4	(0.5–55.7)	1	40	3.0	(0.4–20.9)	1	21	4.8	(0.6–38.7)	28.5*	<0.001	5
Binge eating disorder (hierarchical)	436	17 105	10	141	2.5*	(1.4–4.5)	1	23	1.1	(0.2–8.3)	3	29	7.3*	(2.1–25.8)	1	40	2.0	(0.3–14.7)	2	21	4.8*	(1.0–22.7)	22.7*	<0.001	5
**Any eating disorder**	**598**	**17 105**	**16**	**141**	3.3*	**(1.8–6.0)**	**4**	**23**	7.6*	**(2.4–24.6)**	**4**	**29**	8.3*	**(2.8–24.6)**	**2**	**40**	**2.5**	**(0.6–10.5)**	**3**	**21**	5.5*	**(1.5–20.8)**	43.1*	**<0.001**	5
**Any internalising disorder**	**13 487**	**44 651**	**249**	**332**	9.0*	**(6.5–12.5)**	**36**	**51**	6.0*	**(2.5–14.6)**	**47**	**70**	7.8*	**(4.2–14.4)**	**64**	**110**	4.2*	**(2.4–7.3)**	**39**	**52**	14.8*	**(5.3–41.8)**	287.9*	**<0.001**	5
Attention deficit disorder	589	25 952	42	231	5.7*	(3.3–9.8)	7	38	4.9*	(1.8–13.3)	6	48	4.3*	(1.8–10.0)	5	63	3.5	(0.9–14.1)	1	32	1.5	(0.2–10.6)	54.1*	<0.001	5
Conduct disorder	652	21 666	55	212	7.2*	(4.6–11.3)	10	35	5.9*	(2.5–13.8)	5	37	2.9*	(1.1–7.5)	10	63	5.8*	(2.5–13.5)	4	29	5.1*	(1.7–15.5)	121.2*	<0.001	5
Oppositional defiant disorder	775	18 634	58	186	7.0*	(4.2–11.6)	11	34	6.9*	(2.9–15.9)	7	34	5.1*	(2.2–11.9)	12	48	5.8*	(2.8–12.3)	0	26	0.0*	(0.0–0.0)	12 680.0*	<0.001	5
**Any disruptive behaviour disorder**	**1540**	**28 406**	**100**	**246**	9.1*	**(6.2–13.3)**	**14**	**39**	4.1*	**(1.8–9.1)**	**13**	**49**	5.8*	**(3.1–10.9)**	**19**	**75**	6.7*	**(3.2–14.4)**	**4**	**34**	**1.9**	**(0.6–6.0)**	179.3*	**<0.001**	**5**
Alcohol abuse	4136	42 226	132	305	7.4*	(5.5–9.9)	12	48	2.0	(0.9–4.5)	21	62	4.3*	(2.2–8.5)	34	102	6.9*	(3.7–13.0)	9	48	2.5*	(1.1–5.3)	240.5*	<0.001	5
Alcohol dependence	1345	42 226	68	305	6.8*	(4.6–10.1)	9	48	5.2*	(2.1–13.3)	12	62	5.9*	(2.6–13.3)	16	102	9.2*	(3.9–21.9)	9	48	7.7*	(3.6–16.4)	157.1*	<0.001	5
Drug abuse	1236	40 187	68	294	4.8*	(3.3–7.0)	8	46	3.3*	(1.2–8.6)	5	59	2.3	(0.7–7.8)	17	100	5.7*	(2.7–12.0)	3	45	2.3	(0.7–7.0)	80.6*	<0.001	5
Drug dependence	447	40 187	35	294	5.7*	(3.5–9.1)	5	46	3.5*	(1.2–10.8)	4	59	5.6*	(1.9–16.4)	9	100	8.0*	(3.4–18.7)	3	45	5.4*	(1.6–18.5)	92.5*	<0.001	5
**Any substance use disorder**	**4703**	**42 226**	**145**	**305**	7.5*	**(5.7–10.0)**	**15**	**48**	**2.2**	**(1.0–4.9)**	**25**	**62**	5.6*	**(2.8–11.1)**	**39**	**102**	7.7*	**(4.1–14.2)**	**11**	**48**	2.5*	**(1.2–5.2)**	259.6*	**<0.001**	**5**
**Any externalising disorder**	**5629**	**44 651**	**185**	**332**	9.0*	**(6.8–11.9)**	**22**	**51**	3.0*	**(1.4–6.4)**	**31**	**70**	6.4*	**(3.5–11.6)**	**50**	**110**	9.1*	**(5.0–16.5)**	**13**	**52**	2.2*	**(1.1–4.7)**	339.3*	**<0.001**	**5**
**Any disorder**	**16 324**	**44 651**	**287**	**332**	11.7*	**(7.5–18.2)**	**42**	**51**	6.6*	**(2.0–21.2)**	**50**	**70**	6.0*	**(3.3–11.0)**	**83**	**110**	10.3*	**(5.6–19.1)**	**42**	**52**	14.6*	**(5.2–41.3)**	256.3*	**<0.001**	**5**

Logistic regression was used to compare the prevalence of the comorbid disorder in respondents with IED to that in respondents without IED. All analyses control for participants' age, sex, education (in country-specific quartiles) and country of origin. All tests have 1 df unless otherwise noted. Bold values highlight the results for disorder classes (i,e. groups of disorders).

a Nominator *N* (number of participants reporting the outcome).

b Denominator *N* (number of participants asked the question).

In terms of the pattern of comorbidity, the two subtypes that involved hurting people (‘destroy property and hurt people’ and ‘hurt people only’) had a substantially higher likelihood of an externalising comorbid disorder than the other three subtypes. The least severe subtype (‘threaten people’) was much more likely to have internalising than externalising comorbid disorders, with an especially high odds of social phobia (OR: 13.3; 95% CI: 7.0–25.1). It is also noteworthy that the two subtypes that involved threatening people (‘destroy and threaten’; ‘threaten people’) had the highest odds of bipolar disorder (OR: 10.0; 95% CI: 4.5–22.5 and OR: 11.3; 95% 3.2–39.9, respectively), although confidence intervals around these (and many of the other) estimates are wide.

### Lifetime comorbidity by severity of violence to others

The next analysis (Table 4) subdivided those with IED into two different groups according to the severity of their violence towards others: (i) those who reported that they had hurt someone so badly they needed medical attention and (ii) all the remaining respondents with IED (reference group). Those whose anger attacks resulted in others needing medical attention were both more likely to have disorder comorbidity overall (OR for any disorder: 2.0; 95% CI: 1.0–4.1) and substantially more likely to have disruptive behaviour disorder (OR: 2.7; 95% CI: 1.5–4.8) and substance use disorder (OR: 2.6; 95% CI: 1.6–4.2) comorbidity relative to the reference group whose violence was less severe. Rates of internalising disorders were fairly similar across the two groups.

**Table 4.** Lifetime prevalence of comorbid disorders in respondents who have or have never hurt someone so badly they needed medical attention

	Did not need medical attention				Needed Medical attention				Statistical test				
Comorbid disorder	*n*_1_^a^	*n*_2_^b^	%^c^	s.e.	*n*_1_^a^	*n*_2_^b^	%^c^	s.e.	OR	(95% CI)	Wald X2	*p*-value	df
Agoraphobia (with or without panic)	39	429	8.5	1.5	19	171	9.0	2.3	1.0	(0.5–2.3)	0.0	0.966	1
Generalised anxiety disorder	94	429	21.0	2.4	32	171	22.2	4.3	1.2	(0.7–2.0)	0.3	0.585	1
Panic disorder (with or without agoraphobia)	52	429	9.8	1.5	23	171	9.1	2.4	1.2	(0.6–2.5)	0.3	0.612	1
Post-traumatic stress disorder	67	383	16.3	2.0	32	150	17.8	3.7	1.3	(0.6–2.8)	0.6	0.442	1
Separation anxiety disorder	85	308	26.8	2.7	37	118	33.5	5.0	1.2	(0.7–2.0)	0.3	0.570	1
Social phobia	103	429	24.2	2.4	41	171	23.6	4.1	1.1	(0.6–2.0)	0.2	0.639	1
Specific phobia	108	375	26.5	2.2	44	147	29.0	4.7	1.1	(0.7–1.9)	0.3	0.608	1
**Any anxiety disorder**	**235**	**429**	**54.0**	**2.9**	**101**	**171**	**59.3**	**4.8**	**1.5**	**(0.9–2.4)**	**2.6**	**0.104**	**1**
Major depression or dysthymia (hierarchical)	138	429	27.3	3.0	49	171	26.1	3.9	1.0	(0.6–1.6)	0.0	0.982	1
Bipolar disorder (broad)	59	375	15.4	2.4	39	147	24.1	4.3	1.8*	(1.0–3.3)	4.0*	0.045	1
**Any mood disorder**	**197**	**429**	**40.5**	**3.4**	**88**	**171**	**44.5**	**4.7**	**1.4**	**(0.9–2.2)**	**1.7**	**0.189**	**1**
Bulimia nervosa	9	179	5.3	1.9	3	68	2.7	2.5	0.1	(0.0–3.6)	1.5	0.221	1
Binge eating disorder (hierarchical)	11	179	6.2	2.3	6	68	7.9	2.7	1.1	(0.3–4.3)	0.0	0.874	1
**Any eating disorder**	**20**	**179**	**11.5**	**2.9**	**9**	**68**	**10.6**	**3.7**	**0.6**	**(0.2–2.4)**	**0.4**	**0.514**	**1**
**Any internalising disorder**	**302**	**429**	**67.5**	**2.9**	**125**	**171**	**69.1**	**4.9**	**1.2**	**(0.7–2.0)**	**0.5**	**0.483**	**1**
Attention deficit disorder	37	282	13.9	2.5	23	122	19.3	4.8	1.2	(0.6–2.4)	0.2	0.689	1
Conduct disorder	45	261	16.3	2.2	38	109	41.6	6.9	3.1*	(1.5–6.4)	9.5*	0.002	1
Oppositional defiant disorder	52	228	21.7	3.1	35	95	38.3	6.1	2.0*	(1.0–3.9)	4.4*	0.036	1
**Any disruptive behaviour disorder**	**88**	**304**	**29.0**	**2.8**	**60**	**130**	**48.9**	**5.8**	2.7*	**(1.5–4.8)**	12.0*	**<0.001**	**1**
Alcohol abuse	127	395	31.9	3.0	74	155	52.0	4.6	2.3*	(1.5–3.7)	13.1*	<0.001	1
Alcohol dependence	69	395	16.7	2.1	44	155	29.0	5.3	2.3*	(1.3–4.1)	7.8*	0.005	1
Drug abuse	57	380	13.6	2.2	43	150	26.4	4.3	2.7*	(1.5–4.7)	11.9*	<0.001	1
Drug dependence	35	380	8.7	1.6	21	150	13.0	3.2	1.9	(0.9–3.8)	3.1	0.079	1
**Any substance use disorder**	**144**	**395**	**35.9**	**3.2**	**84**	**155**	**59.0**	**4.8**	2.6*	**(1.6–4.2)**	15.2*	**<0.001**	**1**
**Any externalising disorder**	**189**	**429**	**44.2**	**3.0**	**105**	**171**	**64.8**	**4.7**	2.6*	**(1.6–4.4)**	13.4*	**<0.001**	**1**
**Any disorder**	**344**	**429**	**77.6**	**2.8**	**148**	**171**	**87.7**	**3.0**	**2.0**	**(1.0–4.1)**	**3.4**	**0.064**	**1**

Logistic regression was used to compare the prevalence of the comorbid disorder. All analyses control for participants' age, sex, education (in country-specific quartiles) and country of origin. Bold values highlight the results for disorder classes (i,e. groups of disorders).

a Nominator *N* (number of participants reporting the outcome).

b Denominator *N* (number of participants asked the question).

c Percentages are based on weighted data.

### Suicidality

Lifetime suicidal behaviour amongst the total group with IED was 38.1% (s.e.: 2.0) for ideation, 17.6% (s.e.: 1.6) for plan and 17.4% (s.e.: 1.4) for an attempt (online Supplementary Table S3a). When subdividing total IED into those with and without lifetime comorbidity, suicidal behaviour was much lower among the group without comorbidity, with rates of 26.1% (s.e.: 6.3), 3.5% (s.e.: 1.7) and 6.6% (s.e.: 2.4) for ideation, plans and attempts, respectively, compared with corresponding percentages of 40.9% (s.e.: 2.5), 20.5% (s.e.: 1.9) and 19.4% (s.e.: 1.8) in the group with comorbidity (online Supplementary Table S3b).

There were no statistically significant differences in the prevalence of suicidal behaviour among the five behavioural subtypes (online Supplementary Table S4). When we compared suicidal behaviour between those who did, *v*. those who did not, hurt someone so badly they needed medical attention, the former group reported significantly more suicidal behaviour than the latter group, in all three suicidal behaviour categories (Table 5).

**Table 5.** Lifetime prevalence of suicidality in respondents with IED who have or have not ever hurt someone so badly they needed medical attention

	Respondents who have never hurt someone so badly they needed medical attention				Respondents who have hurt someone so badly they needed medical attention at least once								
	Percentages				Percentages				Parameter estimates				
Suicidality variable	*n*_1_^a^	*n*_2_^b^	%^c^	s.e.	*n*_1_^a^	*n*_2_^b^	%^c^	s.e.	OR	(95% CI)	Wald X2	*p*-value	df
Ideation	169	452	34.9	2.5	87	183	45.0	4.6	1.8*	(1.2–2.9)	7.0	0.008	1
Plan	74	452	14.7	1.8	50	183	24.7	3.4	2.5*	(1.5–4.2)	11.6	<0.001	1
Attempt/gesture	74	452	14.8	1.8	45	183	23.0	3.1	2.3*	(1.4–3.6)	11.8	<0.001	1

Logistic regression was used to compare the prevalence of the suicidality variables. All analyses control for participants' age, sex, education (in country-specific quartiles), and country of origin.

a Nominator *N* (number of participants reporting the outcome).

b Denominator *N* (number of participants asked the question).

c Percentages are based on weighted data.

### Impairment

The proportion of all those with 12-month IED who reported severe functional impairment in at least one domain (home management, work, close relationships or social life) was 39.8% (s.e.: 3.0); with 44.1% (s.e.: 3.7) among those with comorbidity and 17.1% (s.e.: 6.4) among those without comorbidity (data not shown, available on request). The proportion with severe impairment ranged from 30.9% to 44.5% across the five behavioural subtypes (online Supplementary Table S5); although these differences were not statistically significant.

## Discussion

In our first cross-national paper on IED (Scott *et al*., 2016), we examined lifetime prevalence of comorbid mental disorders among those with IED; the present study builds on that earlier report by examining associations (rather than prevalence) of IED with other lifetime disorders and by investigating whether comorbidity patterns vary by IED behavioural subtypes. The two IED subtypes characterised by acts of aggression resulting in harm to others (collectively comprising 73% of those meeting diagnostic criteria with IED) had a greater likelihood of externalising disorder comorbidity than the other three subtypes, although internalising disorder comorbidity was also prevalent. The least destructive subtype ('threaten only’) had a much higher odds of internalising (*v*. externalising) disorder comorbidity and high odds of social phobia in particular. After further subdividing those with IED into perpetrators of more *v*. less violent attacks, we found that the more violent group were more likely to have CD, ODD and substance use disorder comorbidity relative to the less violent group. This more violent group also reported higher rates of lifetime suicidal behaviour (ideation, plans and attempts). There were no significant patterns of variation in functional impairment across IED subtypes.

The major limitations of this study lie in its retrospective assessment of mental disorders. This is known to underestimate lifetime mental disorders (Moffitt *et al*., 2010; Takayanagi *et al*., 2014) and to lead to inaccuracies in the age of onset timing (Simon and Von Korff, 1995). The retrospective method is likely to make it difficult for respondents to determine whether their anger attacks did or did not occur in the context of other disorders. This is a problem because IED is a diagnosis of exclusion, only made once other mental disorders and personality disorders that could better explain the aggressive behaviour have been ruled out. Of the five disorders found most likely to be comorbid with IED in this study (GAD, bipolar disorder, CD, ODD and alcohol dependence), the latter four are either defined by or strongly associated with aggressive behaviour (Jensen *et al*., 2007). This study did exclude from IED diagnosis those who reported that their anger attacks occurred exclusively in the context of depression, substance intoxication or mania, but those with concurrent CD or ODD were not similarly excluded. Moreover, personality disorders were not assessed in enough of the surveys that also assessed IED to assess overlap. As previously reported (Scott *et al*., 2016), a small proportion of the IED sample admitted to purposely torturing or injuring an animal, or arson, within the prior 12 months, so it is possible that these individuals may be more appropriately classified as personality disordered (or CD) than IED.

Some researchers have chosen to deal with the difficulty of differentiating between IED and bipolar disorder by excluding people with a lifetime history of bipolar disorder from the IED sample (Kulper *et al*., 2015; Rynar and Coccaro, 2018; Fahlgren *et al*., 2019). It is unclear why the same theoretical concern does not apply to some of the other comorbid disorders, and if we were to remove all those with lifetime comorbidities associated with impulsive aggression from the group classified with IED we would end up with a much smaller group. It is interesting in this regard to consider the small subgroup of those diagnosed with IED in this study who reported no comorbid disorders. This is a ‘pure’ IED group therefore, whose impulsive anger cannot be attributed to another disorder. We found this group to have much less impairment and suicidality than the bigger group with comorbid disorders. This could suggest that the ramifications of IED for the individual and society are better captured by its comorbid disorders and that the diagnosis of IED *per se* offers little additional information. On the other hand, comorbidity has generally been found to be a marker for severity of psychopathology, and in the case of IED, it may signify that most sufferers experience such persistent tendencies towards irritable temperament and impulsive anger that these tendencies manifest across several diagnostic boundaries.

Our IED behavioural subtypes were defined on the basis of self-reported behaviour and limited to what was available in the CIDI assessment. The subtypes have not been clinically validated and nor was the diagnosis of IED included in the clinical reappraisal studies conducted as part of the World Mental Health surveys (Haro *et al*., 2006). The fact that we did not find variation in suicidal behaviour, or functional impairment, across the five behavioural subtypes suggests that they may not capture clinically useful distinctions. It is noteworthy that the two further approaches to subtyping we report herein did result in significant findings related to suicide. That is, we differentiated between those with and without any lifetime comorbidity, and between those engaging in more or less violent attacks, and we did find suicidality significantly higher among those with comorbidity, and among those engaging in more violent anger attacks that resulted in the victims requiring medical attention. From a clinical point of view therefore, these distinctions rather than the behavioural subtyping distinctions may prove more useful.

The findings of this study are inconsistent with prior clinical studies in several respects. Coccaro et al. who have led the clinical research on IED, have conducted a series of studies in which participants responding to advertisements seeking individuals with anger difficulties were diagnosed with DSM-5 IED, other mental disorders, or no disorders. In these studies, the IED group reported greater functional impairment than the ‘other mental disorder’ comparison group (Kulper *et al*., 2015; Rynar and Coccaro, 2018), whereas our finding of 39.8% of those with IED reporting severe impairment is lower than the corresponding proportion reported for other mental disorders (Scott *et al*., 2018). In these and in other clinical studies from the same group (Fahlgren *et al*., 2019; Fanning *et al*., 2019), IED-associated comorbidity was dominated by internalising disorders, in contrast to our finding that comorbidity was at least equally if not more likely to be with externalising disorders. It is notable that in all of the clinical studies females comprised around half of the IED sample (in contrast to our male-dominated general population sample); this raises the possibility that the clinical findings are influenced by gender, help-seeking or other selection biases.

Our findings illustrate the heterogeneity within the diagnostic category captured by the WMH-CIDI for IED. Depending on how the impulsive anger manifested (in particular, whether it resulted in harm to others), type of comorbidity varied considerably. This variation in comorbidity patterning as a function of whether the anger attacks result in harm to others suggests that the present DSM-5 diagnostic criteria, which allow IED to be defined by either high frequency-less destructive acts or low frequency-more destructive acts, will similarly encompass a population that varies substantially in lifetime comorbidity. In this regard, our study findings are consistent with one study from Coccaro's group, which found that comorbidity patterning varied by DSM-5 IED subtypes (verbal aggression only, physical aggression only, or both) (Look *et al*., 2015). The implications of this heterogeneity in comorbidity for IED as a diagnostic entity are unclear. While it is the case that several mental disorders are characterised by phenotypic subtypes, the findings presented here suggest that these IED behavioural subtypes are characterised by very different patterns of psychopathology over the life course, such that the disorder becomes difficult to classify as internalising or externalising (de Jonge *et al*., 2018).

In conclusion, the findings of this study point to a disparity between the comorbidity patterning and impairment associated with IED in population *v*. clinical studies. This disparity, together with the marked heterogeneity that characterises the diagnostic entity of IED, suggests that it is a disorder that requires much greater research.

## Supplementary material

For supplementary material accompanying this paper visit http://dx.doi.org/10.1017/S2045796020000517. click here to view supplementary material

## Data

The data come from the cross-national World Mental Health Surveys dataset. Due to data-sharing restrictions contained in some individual country agreements with the World Mental Health Surveys Initiative, sharing of the cross-national dataset is not possible.
