# Supplementary material for: Intermittent explosive disorder subtypes in the general population: association with comorbidity, impairment and suicidality
Source: Epidemiol Psychiatr Sci. 2020 Jun 23;29:e138. doi: 10.1017/S2045796020000517 (PMC7327434; doi:10.1017/S2045796020000517)
Supplement: Supplementary file 1 [file S2045796020000517sup001.docx]

| **Table S1. World Mental Health Surveys sample characteristics by World Bank Income categories^a^.** | | | | | | | |
| --- | --- | --- | --- | --- | --- | --- | --- |
| **Country** | **Survey** | **Sample characteristics** | **Field dates** | **Age range^b^** | **Sample Size** | | **Response rate (%)** |
|  |  |  |  |  | **Part 1 sample** | **Part 2 sub-sample** |  |
| **Low - lower middle income countries** | |  |  |  |  |  |  |
| Colombia | NSMH | All urban areas of the country (approximately 73% of the total national population) | 2003 | 18-65 | 4426 | 2381 | 87.7 |
| Iraq | IMHS | Nationally representative. | 2006-7 | 18+ | 4332 | 4332 | 95.2 |
| Nigeria | NSMHW | 21 of the 36 states in the country, representing 57% of the national population. The surveys were conducted in Yoruba, Igbo, Hausa and Efik languages. | 2002-3 | 18+ | 6752 | 2143 | 79.3 |
| Peru | EMSMP | Nationally representative. | 2004-5 | 18-65 | 3930 | 1801 | 90.2 |
| PRC Shen Zhen | Shenzhen | Shenzhen metropolitan area. Included temporary residents as well as household residents. | 2006-7 | 18+ | 7132 | 2475 | 80.0 |
| Ukraine | CMDPSD | Nationally representative. | 2002 | 18+ | 4725 | 1719 | 78.3 |
|  |  |  |  |  |  |  |  |
| **Upper-middle income countries** |  |  |  |  |  |  |  |
| Brazil | São Paulo Megacity | São Paulo metropolitan area. | 2005-7 | 18+ | 5037 | 2942 | 81.3 |
| Bulgaria | NSHS | Nationally representative. | 2003-7 | 18+ | 5318 | 2233 | 72.0 |
| Lebanon | LEBANON | Nationally representative. | 2002-3 | 18+ | 2857 | 1031 | 70.0 |
| Romania | RMHS | Nationally representative. | 2005-6 | 18+ | 2357 | 2357 | 70.9 |
| South Africa | SASH | Nationally representative. | 2003-4 | 18+ | 4315 | 4315 | 87.1 |
|  |  |  |  |  |  |  |  |
| **High-income countries** |  |  |  |  |  |  |  |
| Argentina | AMHES | Eight largest urban areas of the country (approximately 50% of the total national population) | 2015 | 18-98 | 3927 | 2116 | 77.3 |
| Japan | WMHJ | Eleven metropolitan areas. | 2002-6 | 20+ | 4129 | 1682 | 55.1 |
| Northern Ireland | NISHS | Nationally representative. | 2004-7 | 18+ | 4340 | 1986 | 68.4 |
| Poland | EZOP | Nationally representative. | 2010-11 | 18-64 | 10081 | 4000 | 50.4 |
| Portugal | NMHS | Nationally representative. | 2008-9 | 18+ | 3849 | 2060 | 57.3 |
| The United States | NCS-R | Nationally representative. | 2001-3 | 18+ | 9282 | 5692 | 70.9 |
| **Total** |  |  |  |  | **86789** | **45266** |  |
| **Weighted average response rate (%)** | |  |  |  |  |  | **71.1** |
| ^a^ The World Bank. (2008). Data and Statistics. Accessed May 12, 2009 at: http://go.worldbank.org/D7SN0B8YU0 | | | | | | | |
| ^b^For the purposes of cross-national comparisons we limit the sample to those 18+. | | | | | | | |

| **Table S2 Temporal ordering of comorbid disorders and IED.** | | | | | | | | | | | | |
| --- | --- | --- | --- | --- | --- | --- | --- | --- | --- | --- | --- | --- |
|  |  |  |  |  |  |  |  |  |  |  |  |  |
|  | **Comorbid disorder class** | | | | | | | | | | | |
|  | **Internalizing only** | | | | **Externalizing only** | | | | **Internalizing and externalizing** | | | |
| **Temporal ordering** | **n_1_^a^** | **n_2_^b^** | **%^c^** | **SE** | **n_1_^a^** | **n_2_^b^** | **%^c^** | **SE** | **n_1_^a^** | **n_2_^b^** | **%^c^** | **SE** |
| IED before all comorbid disorders | 129 | 435 | 30.6 | 2.9 | 140 | 301 | 49.3 | 3.6 | 134 | 504 | 29.2 | 2.5 |
| IED concurrent with at least one comorbid disorder | 46 | 435 | 9.9 | 1.7 | 22 | 301 | 6.6 | 1.4 | 54 | 504 | 9.9 | 1.6 |
| IED after at least one comorbid disorder | 260 | 435 | 59.5 | 2.9 | 139 | 301 | 44.2 | 3.6 | 316 | 504 | 60.9 | 2.7 |
|  |  |  |  |  |  |  |  |  |  |  |  |  |
| ***Internalizing disorders include GAD, agoraphobia, panic disorder, PTSD, separation anxiety, social phobia, major depression/dysthymia, bipolar disorder, bulimia nervosa, and binge eating disorder.*** | | | | | | | | | | | | |
| ***Externalizing disorders include ADHD, oppositional defiant disorder, conduct disorder, and alcohol and drug abuse and dependence.*** | | | | | | | | | | | | |
| ***^a^Nominator N (number of participants reporting the outcome)*** | | | | | | | | | | | | |
| ***^b^Denominator N (number of participants asked the question)*** | | | | | | | | | | | | |
| ***^c^Percentages are based on weighted data*** | | | | | | | | | | | | |

| **Table S3a. Lifetime prevalence of suicidality in respondents with or without IED.** | | | | | | | | | | | | | |
| --- | --- | --- | --- | --- | --- | --- | --- | --- | --- | --- | --- | --- | --- |
|  |  |  |  |  |  |  |  |  |  |  |  |  |  |
|  | **No IED** | | | | **IED** | | | | | | | | |
|  | **Percentages** | | | | **Percentages** | | | | **Parameter estimates** | | | | |
| **Suicidality variable** | **n_1_^a^** | **n_2_^b^** | **%^c^** | **SE** | **n_1_^a^** | **n_2_^b^** | **%^c^** | **SE** | **OR** | **(95% CI)** | **Wald X2** | **P-value** | **DF** |
| Ideation | 6492 | 86138 | 7.3 | 0.1 | 263 | 651 | 38.1 | 2.0 | 5.4* | (4.5-6.5) | 312.7 | <0.001 | 1 |
| Plan | 2068 | 86138 | 2.3 | 0.1 | 127 | 651 | 17.6 | 1.6 | 5.9* | (4.6-7.4) | 220.7 | <0.001 | 1 |
| Attempt/gesture | 1935 | 86138 | 2.1 | 0.1 | 124 | 651 | 17.4 | 1.4 | 6.3* | (5.0-7.8) | 264.5 | <0.001 | 1 |
|  |  |  |  |  |  |  |  |  |  |  |  |  |  |
| ***Logistic regression was used to compare the prevalence of the suicidality variables. All analyses control for participants' age, sex, education (in country-specific quartiles), and country of origin.*** | | | | | | | | | | | | | |
| ***^a^Nominator N (number of participants reporting the outcome)*** | | | | | | | | | | | | | |
| ***^b^Denominator N (number of participants asked the question)*** | | | | | | | | | | | | | |
| ***^c^Percentages are based on weighted data*** | | | | | | | | | | | | | |

| **Table S3b. Lifetime prevalence of suicidality in respondents with IED with or without any lifetime comorbidity.** | | | | | | | | | | | | | | | | | | | | | | | | | | |  |
| --- | --- | --- | --- | --- | --- | --- | --- | --- | --- | --- | --- | --- | --- | --- | --- | --- | --- | --- | --- | --- | --- | --- | --- | --- | --- | --- | --- |
|  | | | |  | |  | |  | |  |  | |  | |  |  |  |  | | |  | |  | |  | |  |
|  | | | | **Respondents without any lifetime comorbidity** | | | | | | | **Respondents with any lifetime comorbidity** | | | | | | | | | | | | | | | |  |
|  | | | | **Percentages** | | | | | | | **Percentages** | | | | | | **Parameter estimates** | | | | | | | | | |  |
| **Suicidality variable** | | | | **n_1_^a^** | | **n_2_^b^** | | **%^c^** | | **SE** | **n_1_^a^** | | **n_2_^b^** | | **%^c^** | **SE** | **OR** | **(95% CI)** | | | **Wald X2** | | **P-value** | | **DF** | |  |
| Ideation | | | | 23 | | 111 | | 26.1 | | 6.3 | 228 | | 504 | | 40.9 | 2.5 | 1.4 | (0.7-3.1) | | | 0.9 | | 0.340 | | 1 | |  |
| Plan | | | | 5 | | 111 | | 3.5 | | 1.7 | 118 | | 504 | | 20.5 | 1.9 | 5.4* | (1.9-15.4) | | | 10.1 | | 0.001 | | 1 | |  |
| Attempt/gesture | | | | 10 | | 111 | | 6.6 | | 2.4 | 106 | | 504 | | 19.4 | 1.8 | 2.4* | (1.1-5.6) | | | 4.5 | | 0.033 | | 1 | |  |
|  | | | |  | |  | |  | |  |  | |  | |  |  |  |  | | |  | |  | |  | |  |
| ***Logistic regression was used to compare the prevalence of the suicidality variables. All analyses control for participants' age, sex, education (in country-specific quartiles), and country of origin.*** | | | | | | | | | | | | | | | | | | | | | | | | | | |  |
| ***^a^Nominator N (number of participants reporting the outcome)*** | | | | | | | | | | | | | | | | | | | | | | | | | | |  |
| ***^b^Denominator N (number of participants asked the question)*** | | | | | | | | | | | | | | | | | | | | | | | | | | |  |
| ***^c^Percentages are based on weighted data*** | | | | | | | | | | | | | | | | | | | | | | | | | | |  |
| Table S4. **Lifetime prevalence of suicidality in respondents without IED or with specific IED subtypes, with respondents without IED as the reference category.** | | | | | | | | | | | | | | | | | | | | | | | | | | | |
|  |  |  |  | |  |  |  | |  |  |  |  | |  |  |  |  |  |  |  | |  |  |  |  |  | |
|  |  |  | **IED subtypes** | | | | | | | | | | | | | | | | | | | | | | | | |
|  | **No IED** | | **Destroy and hurt** | | | | | | **Destroy and threaten** | | | | | **Destroy things** | | | | **Hurt people** | | | | | **Threaten people** | | | | |
| **Suicidality** | **%^c^** | **SE** | **%^c^** | | **SE** | **OR** | **(95% CI)** | | **%^c^** | **SE** | **OR** | **(95% CI)** | | **%^c^** | **SE** | **OR** | **(95% CI)** | **%^c^** | **SE** | **OR** | | **(95% CI)** | **%^c^** | **SE** | **OR** | **(95% CI)** | |
| Ideation | 7.3 | 0.1 | 43.2 | | 2.8 | 6.3* | (4.9-8.1) | | 44.6 | 8.0 | 7.2* | (3.3-16.0) | | 31.8 | 6.7 | 4.4* | (2.2-8.7) | 28.7 | 4.7 | 4.0* | | (2.6-6.4) | 27.9 | 6.4 | 3.6* | (1.8-7.3) | |
| Plan | 2.3 | 0.1 | 20.4 | | 2.1 | 6.6* | (5.0-8.8) | | 21.3 | 6.1 | 7.5* | (3.5-16.1) | | 8.4 | 3.2 | 2.7* | (1.1-6.2) | 15.2 | 3.3 | 5.7* | | (3.5-9.5) | 12.5 | 4.7 | 4.2* | (1.7-10.4) | |
| Attempt/gesture | 2.1 | 0.1 | 22.1 | | 2.0 | 7.7* | (6.0-10.0) | | 12.2 | 4.8 | 4.1* | (1.5-10.8) | | 10.1 | 3.8 | 3.7* | (1.5-8.8) | 13.0 | 3.1 | 5.5* | | (3.2-9.4) | 12.5 | 4.7 | 4.4* | (1.7-11.4) | |
|  |  |  |  | |  |  |  | |  |  |  |  | |  |  |  |  |  |  |  | |  |  |  |  |  | |
| ***IED subtypes were defined based on behavior during anger attacks (lifetime).*** | | | | | | | | | | | | | | | | | | | | | | | | | | | |
| ***Logistic regression was used to compare the prevalence of the suicidality variables. All analyses control for participants' age, sex, education (in country-specific quartiles), and country of origin.*** | | | | | | | | | | | | | | | | | | | | | | | | | | | |
| ***^c^Percentages are based on weighted data*** | | | | | | | | | | | | | | | | | | | | | | | | | | | |

| Table S5: **Severe 12-month disability in particular domains or in any domain in respondents with specific IED subtypes, with participants who hurt people and destroy things during anger attacks as the reference category.** | | | | | | | | | | | | | | | | | | | | | |
| --- | --- | --- | --- | --- | --- | --- | --- | --- | --- | --- | --- | --- | --- | --- | --- | --- | --- | --- | --- | --- | --- |
|  |  |  |  |  |  |  |  |  |  |  |  |  |  |  |  |  |  |  |  |  |  |
|  | **IED subtypes** | | | | | | | | | | | | | | | | | |  |  |  |
|  | **Destroy and hurt** | | **Destroy and threaten** | | | | **Destroy things** | | | | **Hurt people** | | | | **Threaten people** | | | | **Overall test** | | |
| **Disability domain** | **%^c^** | **SE** | **%^c^** | **SE** | **OR** | **(95% CI)** | **%^c^** | **SE** | **OR** | **(95% CI)** | **%^c^** | **SE** | **OR** | **(95% CI)** | **%^c^** | **SE** | **OR** | **(95% CI)** | **Wald X2** | **P-value** | **DF** |
| Home management | 20.2 | 3.2 | 13.3 | 6.7 | 0.7 | (0.2-2.3) | 9.2 | 4.4 | 0.4 | (0.1-1.2) | 16.6 | 5.5 | 0.6 | (0.2-1.8) | 27.8 | 11.3 | 1.1 | (0.3-4.0) | 3.7 | 0.45 | 4 |
| Ability to work | 16.1 | 2.9 | 11.2 | 6.1 | 0.7 | (0.2-3.0) | 23.5 | 9.7 | 1.5 | (0.5-4.4) | 13.3 | 4.9 | 0.4 | (0.1-1.4) | 12.0 | 6.2 | 0.4 | (0.1-1.6) | 4.0 | 0.40 | 4 |
| Ability to form and maintain close relationships | 31.5 | 4.1 | 19.1 | 7.9 | 0.6 | (0.2-1.7) | 11.8 | 5.1 | 0.3* | (0.1-0.8) | 22.7 | 6.1 | 0.5 | (0.2-1.2) | 23.9 | 8.5 | 0.7 | (0.2-2.3) | 7.1 | 0.13 | 4 |
| Social life | 29.7 | 3.6 | 17.2 | 9.3 | 0.5 | (0.1-1.9) | 14.8 | 6.4 | 0.4 | (0.1-1.3) | 22.1 | 6.5 | 0.6 | (0.2-1.4) | 30.6 | 9.2 | 1.3 | (0.4-4.0) | 5.1 | 0.27 | 4 |
| **Any domain** | **43.1** | **4.0** | **34.0** | **10.5** | **0.8** | **(0.3-2.1)** | **30.9** | **10.1** | **0.6** | **(0.2-1.5)** | **35.1** | **7.4** | **0.7** | **(0.3-1.5)** | **44.5** | **10.7** | **1.5** | **(0.5-4.3)** | **3.7** | **0.44** | **4** |
|  |  |  |  |  |  |  |  |  |  |  |  |  |  |  |  |  |  |  |  |  |  |
| ***IED subtypes were defined based on behavior during anger attacks (lifetime). Severe disability in a particular domain is defined as a score of >6 on the Sheehan Disability Scale for that domain.*** | | | | | | | | | | | | | | | | | | | | | |
| ***Logistic regression was used to compare the prevalence of severe disability. All analyses control for participants' age, sex, education (in country-specific quartiles), and country of origin.*** | | | | | | | | | | | | | | | | | | | | | |
| ***^c^Percentages are based on weighted data*** | | | | | | | | | | | | | | | | | | | | | |
